# *In vitro* potency of novel ertapenem/zidebactam combination (WCK 6777) against carbapenemase-producing Enterobacterales collected across India

**DOI:** 10.1093/jacamr/dlag167

**Published:** 2026-09-07

**Authors:** Yamuna Devi Bakthavatchalam, Robert Bonomo, Adrian Egli, Rosmi Puthan, Abirami Shankar, Soniya Krishnamoorthy, Vasant Nagvekar, Rajni Sabharwal Ekadashi, Sudharsana J, Sneha Rajan, Camilla Rodrigues, Rangineni Jayaprada, Abi Manesh, Balaji Veeraraghavan

**Affiliations:** Department of Clinical Microbiology, Christian Medical College, Vellore, India; Department of Medicine, Case Western Reserve University School of Medicine, Cleveland, OH, USA; Institute of Medical Microbiology, University of Zurich, Zurich, Switzerland; Department of Medicine, Case Western Reserve University School of Medicine, Cleveland, OH, USA; Department of Clinical Microbiology, Christian Medical College, Vellore, India; Department of Clinical Microbiology, Christian Medical College, Vellore, India; Department of Infectious Disease, Lilavati Hospital & Research Centre, Mumbai, India; Department of Microbiology, Mahatma Gandhi Medical College & Hospital, Puducherry, India; Department of Microbiology, Baby Memorial Hospital, Kozhikode, India; Department of Microbiology, Narayani Hospital and Research Centre, Vellore, India; Department of Microbiology, P. D. Hinduja Hospital and Medical Research Centre, Mumbai, India; Department of Microbiology, Sri Venkateswara Institute of Medical Sciences, Tirupati, India; Department of Infectious Disease, Christian Medical College, Vellore, India; Department of Clinical Microbiology, Christian Medical College, Vellore, India

## Abstract

**Background and objectives:**

Ertapenem is well suited for outpatient parenteral antimicrobial therapy (OPAT) due to its once-daily dosing and favourable safety profile; however, increasing enzyme-mediated resistance limits its clinical utility. Ertapenem/zidebactam (WCK 6777) is a novel β-lactam/β-lactam enhancer combination in which zidebactam exhibits high-affinity binding to penicillin-binding protein 2 (PBP2), augmenting ertapenem activity and overcoming β-lactamase-mediated resistance. We evaluated the *in vitro* activity of ertapenem/zidebactam against carbapenem-resistant *Escherichia coli* and *Klebsiella pneumoniae* clinical isolates from India.

**Methods:**

MICs of ertapenem/zidebactam and comparator agents (carbapenems, imipenem/relebactam, ceftazidime/avibactam and aztreonam/avibactam) were determined by broth microdilution method. Carbapenemase genes and PBP3 insertions were identified using PCR.

**Results:**

Ertapenem/zidebactam demonstrated potent activity against *E. coli*, inhibiting 99.5% of isolates at the PK/PD breakpoint of ≤8 mg/L (MIC_90_, 0.5 mg/L), including NDM producers with PBP3 insertions. Against *K. pneumoniae*, susceptibility reached 93.6% among NDM producers and 88.5% among dual carbapenemase producers at the same breakpoint. Comparator agents showed variable activity, with limited efficacy against metallo-β-lactamase producers.

**Conclusions:**

The enhanced activity of ertapenem/zidebactam is attributed to zidebactam’s dual mechanism, β-lactamase inhibition and high-affinity PBP2 binding, enabling effective coverage despite complex resistance mechanisms. Its once-daily dosing supports its potential as an OPAT-enabling therapeutic option and warrant further clinical development.

## Introduction

Enterobacterales, particularly *Escherichia coli* and *Klebsiella pneumoniae*, are the frequent causes of community-acquired Gram-negative infections. Carbapenemases, once largely confined to nosocomial Gram-negative pathogens, are increasingly detected in community isolates of these species, representing a growing public health challenge.^1^ Outpatient infections include complicated urinary tract infection, complicated intra-abdominal infection, diabetic foot infection and bone and joint infection. Importantly, many patients with community-acquired carbapenemase-producing Enterobacterales (CPE) infection are clinically stable at presentation or achieve early haemodynamic stability and source control during admission. In such cases, use of once-daily CPE-active agents suitable for outpatient parenteral antimicrobial treatment (OPAT) could avoid hospitalization or facilitate early discharge through transition to home or outpatient-based intravenous treatment.^2^ OPAT is an effective modality for managing such infections in outpatient settings.^2,3^

Several recently approved intravenous agents are active against serine CPE (ceftazidime/avibactam, meropenem/vaborbactam and imipenem/relebactam) or both serine and metallo-β-lactamase (MBL) producing isolates (cefiderocol and aztreonam/avibactam) Enterobacterales.^4^ However, spectrum gaps continue to exist; for example, cefiderocol and aztreonam/avibactam exhibit limited activity against NDM-producing *E. coli* harbouring four amino acid insertion in PBP3.^5,6^ Moreover, although their spectrum is relevant for outpatient care, their dosing regimens is not OPAT enabling. Frequent dosing (every 6 to 8 h) and prolonged infusion times limit their outpatient use and some agents lack the physico-chemical properties required for longer infusion in this setting.

The lack of OPAT-enabling antibiotics contributes to unnecessary or prolonged hospitalization, increased healthcare resource use, cost and a higher risk of hospital-associated complications. To address this gap, ertapenem/zidebactam is in clinical development as a once-daily CPE-active combination with pharmacokinetic/pharmacodynamic (PK/PD) properties providing broad coverage against both serine and MBL producing Enterobacterales.^7^ Zidebactam is a bicyclo-acyl hydrazide diazabicyclooctane derivative with dual functionality: potent β-lactamase inhibition (class A and class C enzymes) and intrinsic antibacterial activity mediated through high-affinity binding to penicillin-binding protein 2 (PBP2).^8^ Inhibition of PBP2 induces spheroplast formation that is maintained despite β-lactamase expression, owing to zidebactam’s non-β-lactam structure, which imparts resistance to enzymatic hydrolysis.^9^

Zidebactam has been combined with both cefepime and ertapenem, and in each case conferring activity against both serine- and metallo-carbapenemase producers, through complementary inhibition of PBP2 and PBP3, an approach associated with enhanced bactericidal activity compared with single-PBP inhibition.^8,10^ Cefepime/zidebactam combination has been recently approved by United States Food and Drug Administration. In combination with ertapenem, zidebactam overcomes key limitations of ertapenem monotherapy, including low susceptibility breakpoints, reduced activity due to impermeability in ESBL- and AmpC-producing organisms, and vulnerability to carbapenemase-mediated hydrolysis. These features make ertapenem/zidebactam a rational candidate for OPAT treatment of CPE infections.

Recently, ertapenem/zidebactam (WCK 6777) completed a Phase 1 study evaluating the safety and tolerability of multiple ascending once-daily doses (1 + 1 g to 3 + 3 g). In addition, translational studies in neutropenic murine infection model showed that a human-simulated regimen of 2 + 2 g once-daily achieved robust bactericidal activity, exceeding the translational efficacy threshold of a 1-log_10_ CFU reduction, against CPE with MICs up to 8 mg/L.^8^ As this combination, advances in clinical development, we evaluated its *in vitro* activity against a well-characterized collection of CPE isolates recovered across India.

## Methods

### Bacterial isolates

The study panel comprised carbapenemase-producing *E. coli* (*n* = 235) and *K. pneumoniae* (*n* = 252) isolates collected during 2024 to 2025 from nine tertiary-care hospitals across India. We consecutively obtained isolates from bloodstream infections, skin and soft tissue infections, urinary tract infections and pneumonia, with only one isolate included per patient episode. We performed species identification using MALDI-TOF MS (bioMérieux, Marcy-l’Étoile, France).

### Antimicrobial susceptibility testing

We determined the minimum inhibitory concentrations (MICs) for ertapenem, aztreonam/avibactam, ceftazidime/avibactam and imipenem/relebactam by reference broth microdilution method according to the Clinical and Laboratory Standards Institute (CLSI) guideline M07.^11^ We determined MICs of ertapenem/zidebactam (both components supplied by Wockhardt, Aurangabad, India) at a fixed 1:1 ratio. Avibactam was tested at a fixed concentration of 4 mg/L in combination with aztreonam or ceftazidime, and relebactam at a fixed concentration of 4 mg/L with imipenem. We obtained comparator agents from MedChemExpress (Monmouth Junction, NJ, USA). We determined all MICs in parallel using the same bacterial inoculum. We applied a PK/PD breakpoint of ≤8 mg/L for interpretation of ertapenem/zidebactam susceptibilty,^7^ whereas comparator agents were interpreted according to CLSI breakpoints (M100, 35th edition).^12^ We performed quality control testing concurrently using *E. coli* ATCC 25922, *K. pneumoniae* ATCC 700603, *K. pneumoniae* JM 3 (internal quality control strain) and ATCC BAA-1705.

### *Genotypic detection of carbapenemases and PBP3 insertion in* E. coli

We screened all isolates with imipenem and/or meropenem MICs ≥2 mg/L for carbapenemase genes (KPC, IMP, VIM, NDM and OXA-48–like). In addition, we screened all carbapenemase-producing *E. coli* isolates for PBP3 insertions by mismatch amplification mutation assay (MAMA) PCR, as previously described.^5^

## Results

The MIC distributions of ertapenem/zidebactam and comparator agents against carbapenemase-producing-*E. coli* and -*K. pneumoniae* are shown in Table 1. Among *E. coli* isolates, 83% produced *bla*_NDM_, 11% co-carried *bla*_NDM_ and *bla*_OXA-48–like_ carbapenemases and 5% carried bla_OXA-48–like_ enzymes alone. All tested *E. coli* isolates (235/235, 100%) harboured a four-amino-acid PBP3 insertion, irrespective of carbapenemase genotype. Among *K. pneumoniae* isolates (Table 2), 43% carried *bla*_OXA-48–like_ carbapenemases, 38% co-carried *bla*_NDM_ and *bla*_OXA-48–like_ enzymes, and 19% carried *bla*_NDM_.

**Table 1.** MIC distribution of ertapenem/zidebactam and comparator agents against carbapenemase-producing *E. coli* carrying PBP3 insertions (*n* = 235)

Antibiotic	No. of isolates with MIC (mg/L) [% Cumulative inhibition]														Percentage S^a^ (CLSI)	MIC (mg/L)	
	≤0.03	0.06	0.12	0.25	0.5	1	2	4	8	16	32	64	128	>128		MIC_50_	MIC_90_
NDM + PBP3 insert (*n* = 195)																	
Ertapenem									*1* *[0.5]*		*7* *[4.1]*	*36* *[22.6]*	*90* *[68.7]*	*61* *[100]*	0	128	>128
Ertapenem/zidebactam	36 [18.5]	20 [28.7]	59 [59]	54 [86.7]	21 [97.4]	4 [99.5]					*1* *[100]*				99.5	0.12	0.5
Meropenem							*1* *[0.5]*	*2* *[1.5]*	*2* *[2.6]*	*3* *[4.1]*	*15* *[11.8]*	*54* *[39.5]*	*95* *[88.2]*	*23* *[100]*	0	128	>128
Imipenem						1 [0.5]	*1* *[1.0]*		*3* *[2.56]*	*43* *[24.6]*	*86* *[68.7]*	*41* *[89.7]*	*18* *[99]*	*2* *[100]*	0.5	32	128
Imipenem/relebactam						1 [0.5]	*1* *[1.0]*	*1* *[1.5]*	*6* *[4.6]*	*48* *[29.2]*	*80* *[70.3]*	*42* *[91.8]*	*15* *[99.5]*	*1* *[100]*	0.5	32	64
Ceftazidime/avibactam										*1* *[0.5]*	*5* *[3.1]*	*7* *[6.7]*	*16* *[14.9]*	*166* *[100]*	0	>128	>128
Aztreonam/avibactam	5 [2.6]	4 [4.6]		4 [6.7]	14 [13.9]	27 [27.7]	38 [47.2]	36 [65.6]	*41* *[86.7]*	*24* *[99]*	*2* *[100]*				65.6	4	16
NDM + OXA-48 like + PBP3 insert (*n* = 27)																	
Ertapenem										*1* *[3.7]*	*[3.7]*	*4* *[18.5]*	*10* *[55.6]*	*12* *[100]*	0	128	>128
Ertapenem/zidebactam	6 [22.2]		8 [51.9]	10 [88.9]	1 [92.6]		1 [96.3]					*1* *[100]*			96.3	0.12	0.25
Meropenem									*1* *[3.7]*		*3* *[14.8]*	*9* *[48.2]*	*10* *[85.2]*	*4* *[100]*	0	128	>128
Imipenem							*1* *[3.7]*			*6* *[25.9]*	*5* *[44.4]*	*12* *[88.9]*	*2* *[96.3]*	*1* *[100]*	0	64	64
Imipenem/relebactam							*1* *[3.7]*		*2* *[11.1]*	*4* *[25.9]*	*8* *[55.6]*	*9* *[88.9]*	*2* *[96.3]*	*1* *[100]*	0	32	64
Ceftazidime/avibactam										*1* *[3.7]*	*1* *[7.41]*	*1* *[11.1]*	*1* *[14.8]*	*23* *[100]*	0.00	>128	>128
Aztreonam/avibactam	3 [11.1]			1 [14.8]	1 [18.5]	2 [25.9]	3 [37.0]	7 [63]	*5* *[81.5]*	*2* *[88.9]*	*1* *[92.6]*	*1* *[96.3]*		*1* *[100]*	63	4	16
OXA-48 like + PBP3 insert (*n* = 13)																	
Ertapenem					2 [15.4]		*2* *[30.8]*	*4* *[61.5]*	*3* *[84.6]*	*2* *[100]*					15.4	4	16
Ertapenem/zidebactam	3 [23.1]		5 [61.5]	4 [92.3]		1 [100]									100	0.12	0.25
Meropenem			2 [15.4]	1 [23.1]	6 [69.2]	3 [92.3]		*1* *[100]*							92.3	0.5	1
Imipenem			1 [7.7]		4 [38.5]	2 [53.9]	*4* *[84.6]*	*1* *[92.3]*	*1* *[100]*						53.9	1	4
Imipenem/relebactam	1 [7.7]		1 [15.4]	4 [46.2]	2 [61.5]	1 [69.2]	*3* *[92.3]*	*1* *[100]*							69.2	0.5	2
Ceftazidime/avibactam	3 [23.1]			1 [30.8]		3 [53.8]	4 [84.6]	2 [100]							100	1	4
Aztreonam/avibactam	3 [23.1]			1 [30.8]	1 [38.5]		1 [46.2]	2 [61.5]		*5* *[100]*					61.5	4	16

^a^Percentage susceptibility interpreted according to CLSI, *M100, Thirty-Fifth Edition*, 2025.

Italicized text: non-susceptible MIC range according to CLSI interpretive criteria.

**Table 2.** MIC distribution of ertapenem/zidebactam and comparator agents against carbapenemase-producing *K. pneumoniae* (*n* = 252)

Antibiotic	No. of isolates with MIC (mg/L) [% Cumulative inhibition]													Percentage S^a^ (CLSI)	MIC (mg/L)	
	≤0.06	0.12	0.25	0.5	1	2	4	8	16	32	64	128	>128		MIC_50_	MIC_90_
NDM producers (*n* = 47)																
Ertapenem									*3* *[6.4]*	*6* *[19.2]*	*8* *[36.2]*	*15* *[68.1]*	*15* *[100]*	0	128	>128
Ertapenem/zidebactam	3 [6.4]	7 [21.3]	8 [38.3]	7 [53.2]	7 [68.1]	5 [78.7]	4 [87.2]	3 [93.6]	*2* *[97.9]*			*1* *[100]*		93.6	0.5	8
Meropenem							*1* *[2.1]*		*2* *[6.4]*	*6* *[19.2]*	*9* *[38.3]*	*18* *[76.6]*	*11* *[100]*	0	128	>128
Imipenem								*5* *[10.6]*	*7* *[25.5]*	*10* *[46.8]*	*12* *[72.3]*	*10* *[93.6]*	*3* *[100]*	0	64	128
Imipenem/relebactam								*6* *[12.8]*	*6* *[25.5]*	*13* *[53.2]*	*9* *[72.3]*	*12* *[97.9]*	*1* *[100]*	0	32	128
Ceftazidime/avibactam												*2* *[4.3]*	*45* *[100]*	0	>128	>128
Aztreonam/avibactam	21 [44.7]	17 [80.9]	7 [95.7]	1 [97.9]		1 [100]								100	0.12	0.25
NDM plus OXA-48-like producers (*n* = 96)																
Ertapenem									*1* *[1.04]*	*3* *[4.17]*	*2* *[6.25]*	*15* *[21.88]*	*75* *[100]*	0	>128	>128
Ertapenem/zidebactam	2 [2.1]		9 [11.5]	17 [29.2]	19 [49]	22 [71.9]	14 [86.5]	2 [88.5]	*7* *[95.8]*	*4* *[100]*				88.5	2	16
Meropenem								*3* *[3.1]*	*1* *[4.2]*	*3* *[7.3]*	*13* *[20.8]*	*25* *[46.9]*	*51* *[100]*	0	>128	>128
Imipenem					2 [2.1]			*6* *[8.3]*	*4* *[12.5]*	*7* *[19.8]*	*23* *[43.8]*	*30* *[75]*	*24* *[100]*	2.08	128	>128
Imipenem/relebactam					2 [2.1]		*3* *[5.2]*	*2* *[7.3]*	*3* *[10.4]*	*12* *[22.9]*	*14* *[37.5]*	*45* *[84.4]*	*15* *[100]*	2.08	128	>128
Ceftazidime/avibactam												*1* *[1.04]*	*95* *[100]*	0	>128	>128
Aztreonam/avibactam	11 [11.46]	34 [46.88]	37 [85.42]	9 [94.79]	2 [96.88]		2 [98.96]		*1* *[100]*					98.96	0.25	0.5
OXA-48-like producers (*n* = 109)																
Ertapenem				1 [0.92]	*2* *[2.75]*	*6* *[8.26]*	*7* *[14.68]*	*4* *[18.35]*		*8* *[25.69]*	*23* *[46.79]*	*40* *[83.49]*	*18* *[100]*	0.92	128	>128
Ertapenem/zidebactam	1 [0.92]	2 [2.75]	10 [11.93]	12 [22.94]	49 [67.89]	30 [95.41]	2 [97.25]	3 [100]						100	1	2
Meropenem			4 [3.67]	7 [10.09]	5 [14.68]	*2* *[16.51]*	*4* *[20.18]*	*13* *[32.11]*	*31* *[60.55]*	*35* *[92.66]*	*8* *[100]*			14.68	16	32
Imipenem		1 [0.92]	3 [3.67]	6 [9.17]	5 [13.76]	*24* *[35.78]*	*40* *[72.48]*	*20* *[90.83]*	*3* *[93.58]*	*4* *[97.25]*	*3* *[100]*			13.76	4	8
Imipenem/relebactam	1 [0.92]	4 [4.59]	8 [11.93]	3 [14.68]	11 [24.77]	*43* *[64.22]*	*23* *[85.32]*	*8* *[92.66]*	*5* *[97.25]*	*3* *[100]*				24.77	2	8
Ceftazidime/avibactam	1 [0.92]	2 [2.75]	12 [13.76]	56 [65.14]	29 [91.74]	8 [99.08]	1 [100]							100	0.5	1
Aztreonam/avibactam	22 [20.2]	41 [57.8]	35 [89.9]	9 [98.2]	1 [99.1]	1 [100]								100	0.12	0.25

^a^Percentage susceptibility interpreted according to CLSI, *M100, Thirty-Fifth Edition*, 2025.

Italicized text: non-susceptible MIC range according to CLSI interpretive criteria.

In *E. coli*, ertapenem/zidebactam showed potent activity against NDM producers, inhibiting all but one isolate at MICs ≤1 mg/L. Using an ertapenem breakpoint of ≤0.5 mg/L, 97.4% of isolates were inhibited, increasing to 99.5% at the PK/PD breakpoint of 8 mg/L (MIC_90_, 0.5 mg/L). Among isolates co-producing NDM and OXA-48–like carbapenemases, inhibition increased from 92.3% at ≤0.5 mg/L to 96.3% at 8 mg/L (MIC_90_, 0.25 mg/L). All OXA-48–like producing isolates were inhibited at MICs ≤1 mg/L. By comparison, aztreonam/avibactam susceptibility was 64.6% among NDM producers and 62.3% for dual NDM plus OXA-48–like producers. Within the OXA-48-like subgroup, ceftazidime/avibactam inhibited 100% of isolates at MICs ≤4 mg/L, whereas aztreonam/avibactam and imipenem/relebactam inhibited 61.5% and 69.2%, respectively. As expected, ceftazidime/avibactam and imipenem/relebactam lacked activity against NDM-producing *E. coli* (Table 1).

In *K. pneumoniae*, ertapenem/zidebactam inhibited 53.1% of NDM-producing isolates at ≤0.5 mg/L, increasing to 93.6% at ≤8 mg/L. The activity was relatively lower against isolates co-producing NDM and OXA-48–like carbapenemases, with 29.1% inhibited at ≤0.5 mg/L and 88.5% at ≤8 mg/L. Ertapenem/zidebactam MICs were broadly distributed from ≤0.06 to 8 mg/L, with most NDM producers at 0.12–1 mg/L, dual carbapenemase producers at 0.5–2 mg/L and OXA-48–like isolates at 1–2 mg/L. Aztreonam/avibactam inhibited all but one *K. pneumoniae* isolate, irrespective of carbapenemase genotype. Ceftazidime/avibactam and imipenem/relebactam were inactive against NDM producers. Among OXA-48–like producing isolates, ertapenem/zidebactam inhibited 92.3% at ≤0.5 mg/L and 100% at ≤8 mg/L, as expected ceftazidime/avibactam also inhibited all isolates at MICs ≤4 mg/L (MIC_90_, 1 mg/L) (Table 2).

## Discussion

In Enterobacterales, the convergence of extended-spectrum β-lactamases (ESBLs), AmpC β-lactamases, porin loss and carbapenemases has progressively eroded the clinical utility of ertapenem, despite its established value for OPAT.^13^ Restoring ertapenem activity is therefore of considerable clinical interest. In this context, combining ertapenem with the β-lactam enhancer zidebactam is a rational strategy. In the present study, ertapenem/zidebactam showed potent *in vitro* activity against *E. coli* and *K. pneumoniae* isolates producing MBLs and/or OXA-48-like carbapenemases. At the PK/PD breakpoint of 8 mg/L, the combination inhibited >90% of isolates overall, indicating substantial activity against phenotypes typically resistant to ertapenem alone.

This activity cannot be explained by β-lactamase inhibition alone. Zidebactam, combines inhibition of class A and class C β-lactamases with intrinsic antibacterial activity through high-affinity binding to PBP2.^8^ Notably, nearly all *E. coli* isolates including NDM producers, carried PBP3 insertion, a mechanism associated with reduced susceptibility to several β-lactam/β-lactamase inhibitor combinations including aztreonam/avibactam. Despite this, ertapenem/zidebactam retained high activity, suggesting that strong PBP2 engagement can overcome the functional impact of PBP3 insertions.^7,8^

In *K. pneumoniae*, activity is best explained by synergistic inhibition of multiple PBPs. Although zidebactam does not inhibit MBLs or OXA-48-like carbapenemases, which efficiently hydrolyse ertapenem, the combination remained active against such isolates. This supports a mechanism in which zidebactam’s binding to PBP2, complements ertapenem activity mediated through targeting other PBPs, preserving antibacterial efficacy despite carbapenemase-mediated hydrolysis.^7,14^ Notably, even recently developed broad-spectrum β-lactamase inhibitors such as xeruborbactam, designed to inhibit both serine β-lactamases and MBLs, have demonstrated only limited activity against NDM-producing Enterobacterales.^15^ We propose that the synergistic inhibition of cell wall synthesis (PBP2) and the inhibitory activity of zidebactam allows more ertapenem to enter the cell and overcome β-lactamase activity.

The clinical relevance of these findings should be considered in light of PK/PD data. Although the ertapenem susceptibility breakpoint is 0.5 mg/L, translational animal models have shown efficacy of ertapenem/zidebactam at MICs up to 8 mg/L.^7^ This expanded activity likely reflects a reduced %*f*T > MIC requirement for ertapenem in the presence of zidebactam, consistent with previous observations for cefepime/zidebactam.^7,16,17^ The high proportion of isolates inhibited at the 8 mg/L in the present study is therefore consistent with the enhanced pharmacodynamic profile of the combination.^14^ Taken together, the broad coverage of CPE combined with ertapenem’s once-daily dosing and established suitability for OPAT supports continued clinical development of ertapenem/zidebactam as a promising therapeutic option for CPE infections.

### Conclusion

Ertapenem/zidebactam exhibited potent *in vitro* activity against NDM-producing *E. coli* with PBP3 inserts, NDM-producing *K. pneumoniae* including dual carbapenemase producers, and OXA-48 like producers. Its once-daily dosing and spectrum against Enterobacterales is suited for OPAT. These findings position ertapenem/zidebactam as a focused OPAT option for MDR Enterobacterales and support its continued clinical development for infections caused by organisms with diverse β-lactamases-mediated resistance.
